# Editorial: Personalized management of acromegaly

**DOI:** 10.3389/fendo.2023.1214152

**Published:** 2023-05-19

**Authors:** Nicholas A. Tritos

**Affiliations:** Neuroendocrine Unit and Neuroendocrine and Pituitary Tumor Clinical Center, Massachusetts General Hospital and Harvard Medical School, Boston, MA, United States

**Keywords:** acromegaly, pituitary adenoma, medical therapy, surgery, radiation therapy, growth hormone

Acromegaly is a systemic disorder that results from chronic exposure to excess growth hormone (GH), generally as a consequence of autonomous GH secretion from a somatotroph pituitary adenoma (1). Acromegaly is associated with many comorbidities and carries an increased mortality risk, which is blunted in patients who achieve biochemical control of their disease (2).

The present Research Topic (“*Personalized management of acromegaly*”) includes 6 articles that review important aspects of acromegaly pertaining to disease pathophysiology, comorbidities and management and highlight current uncertainties and challenges in the field.

Freda comprehensively reviews abnormalities in distribution of adipose tissue in patients with acromegaly, who are insulin resistant despite a decrease in visceral adiposity. This phenomenon appears to be associated with ectopic lipid deposition in skeletal muscle. She elegantly reviews putative factors that may influence the distribution of adipose tissue in patients with acromegaly and highlights recent research observations, including the possible role of agouti related protein, ghrelin and adipokines.

Kasuki et al. thoughtfully discuss available data on the epidemiology and pathophysiology of colorectal neoplasia in patients with acromegaly. This is an area of active controversy, wherein some studies, but not others, have reported an increase in incidence of colorectal cancer (3). Several potential methodological limitations may account for such discrepancies, as the authors comprehensively discuss. Of note, a recent meta-analysis of 23 studies (9677 patients) reported an increase in the standardized incidence ratio (SIR) of colorectal cancer in comparison with the general population [SIR: 2.6 (1.7 to 4.0)] (4). As a corollary, screening (surveillance) colonoscopies have been recommended in patients with acromegaly by several professional societies, albeit with substantial variations with regards to the time of onset and the frequency of screening.

Four additional articles review salient aspects of management of patients with acromegaly. Bray et al. comprehensively discuss the role of transsphenoidal surgery in acromegaly, which represents the cornerstone of management in the vast majority of patients. They review the surgical approach, preoperative and postoperative management and discuss predictors of remission.

Labadzhyan and Melmed thoughtfully review molecular targets for current medical therapies for acromegaly (somatostatin receptor ligands, dopamine agonists and growth hormone receptor antagonists) as well as a host of molecules involved in the regulation of cell cycle and growth hormone secretion, which may eventually serve as targets for novel therapies. They also discuss possible predictors of therapeutic response to medical therapies, which may afford a personalized (“precision medicine”) approach to medical management.

In an elegant review, Liu and Fleseriu discuss modalities used to deliver radiation therapy to patients with acromegaly, review the role, effectiveness and toxicities associated with this treatment, while highlighting uncertainties and future directions in the field. This is another evolving area wherein ongoing technical advances and further research will continue to shape our views regarding the indications and role of this important treatment modality.

Bianchi et al. thoughtfully discuss the problem of the aggressive somatotroph adenoma, which represents a major challenge in management and necessitates a multidisciplinary approach at a pituitary center, involving the role of surgery, radiation therapy, and medical therapy, such as temozolomide, and review novel, emerging therapies. This is also an area where further research is needed to identify pertinent mechanisms driving tumorigenesis and develop rationally developed, novel therapies based on emerging molecular targets.

The information presented in the 6 articles of this Research Topic highlights many of the complexities involved in the pathophysiology and management of acromegaly and supports the promulgation of “Pituitary Tumor Centers of Excellence”, which will serve to advance both research and the care of patients with pituitary disorders, including acromegaly (5).

The editors of the present Research Topic are deeply indebted to our colleagues, all experts in the field, who authored these remarkable articles as well as the reviewers who provided helpful comments and suggestions. The editors believe that the information presented in this Research Topic will serve as a thoughtful introduction to important aspects of the pathophysiology, comorbidities and management of patients with acromegaly and will help stimulate the readers’ interest in reviewing the primary literature.

## Author contributions

The author confirms being the sole contributor of this work and has approved it for publication.
